# RELATIONSHIP BETWEEN CEREBRAL MICROINFARCTS AND DEMENTIA BY GENDER AMONG COMMUNITY-BASED OLDER ADULTS

**DOI:** 10.1093/geroni/igad104.1271

**Published:** 2023-12-21

**Authors:** Mo-kyung Sin, Yan Cheng, Jeffrey Roseman, Edward Zamrini, Ali Ahmed

**Affiliations:** Seattle University, Seattle, Washington, United States; George Washington University, Washington, District of Columbia, United States; University of Alabama at Birmingham, Birmingham, Alabama, United States; Irvine Clinical Research, Irvine, California, United States; Washington DC Veterans Medical Center, Washington, District of Columbia, United States

## Abstract

Cerebral microinfarcts are common in older adults and associated with cognitive impairment. Less is known about sex-related variation in the relationship between microinfarcts and dementia in older adults, the examination of which was the objective of this study. This case-control study was based on 727 participants (308 men, 419 women) in the Adult Changes in Thought (ACT) autopsy data. Microinfarcts (presence, number, and location) were ascertained by blinded board-certified neuropathologists, and dementia diagnoses (yes, no) were made by the ACT Consensus Diagnosis Conference per DSM-IV criteria. Multivariable logistic regression analyses were conducted to examine the relationship. Microinfarcts were present in 356 (49%) participants (144 had one; 211 ≥2, 257 cortical, 216 subcortical), and 381 (52.4%) had dementia. More participants with dementia had ≥2 microinfarcts, although there was no difference in the distribution of a single microinfarct. Adjusted OR (95% CI) for dementia associated with any microinfarct was 1.36 (0.97–1.91). Respective ORs (95% CIs) in women and men were 1.45 (0.91–2.30) and 1.24 (0.75–2.06; p for interaction, 0.34). Adjusted OR (95% CI) for dementia associated with cortical and subcortical microinfarcts were 1.19 (0.83–1.69) and 1.65 (1.14–2.38), respectively, without any evidence of heterogeneity (p for interaction, 0.96 and 0.55, respectively). The presence of subcortical microinfarcts, but not cortical, had a significant association with dementia in older adults, but there was no evidence of a sex-related heterogeneity in that association. Future studies need to examine strategies to delay and manage vascular risk factors contributing to subcortical microinfarcts.

